# Calciphylaxis in End-Stage Renal Disease: A Rare Condition With High Mortality

**DOI:** 10.7759/cureus.26752

**Published:** 2022-07-11

**Authors:** Ramya Bachu, Tanvi H Patel, Stefan Hemmings

**Affiliations:** 1 Internal Medicine, Baptist Health-University of Arkansas for Medical Sciences (UAMS), North Little Rock, USA; 2 Nephrology, Baptist Health-University of Arkansas for Medical Sciences (UAMS), North Little Rock, USA

**Keywords:** end-stage renal disease (esrd), calciphylaxis, dialysis, wound care, mortality

## Abstract

Calciphylaxis is a rare but life-threatening condition, seen in patients with end-stage renal disease (ESRD) on renal replacement therapy. Its pathogenesis is not completely known, but microvascular calcification and thrombosis are considered the likely processes. It is characterized by significant morbidity due to severe pain and nonhealing wounds with frequent hospitalizations. Sepsis is the most common cause of mortality with more than 50% of patients dying within the first year after diagnosis. Optimal management requires a multidisciplinary approach. We describe a case of a 66-year-old female with ESRD on hemodialysis (HD) who presented with severe progressive calciphylaxis wounds on both lower extremities and died within two months after diagnosis. She had multiple admissions in the past for cellulitis when she presented with swelling in the legs and chronic wounds. Our goal is to increase awareness among physicians to include calciphylaxis in their differential diagnosis when treating ESRD patients with significant risk factors to detect it early and prevent morbidity and mortality.

## Introduction

Calciphylaxis or calcific uremic arteriolopathy is a rare but life-threatening condition that is characterized by painful skin lesions including indurated plaques, livedo reticularis, and necrosis that can progress to penetrating ulceration with secondary wound infection. It is associated with high morbidity and mortality, with a reported one-year mortality of 30-80% [1]. Most commonly, it affects patients with end-stage renal disease (ESRD) and can occur in both peritoneal and hemodialysis (HD) patients. Risk factors include hyperphosphatemia, hypercalcemia, hyper- and hypoparathyroidism, hypercoagulability, diabetes mellitus, female sex, white race, autoimmune diseases, obesity, and warfarin use [2]. Here, we describe a case of a 66-year-old female with ESRD on hemodialysis (HD) who presented with severe progressive calciphylaxis wounds on lower extremities and died within two months after diagnosis. The purpose of this case report was to increase awareness among physicians to include calciphylaxis in their differential diagnosis while treating ESRD patients since this condition is associated with high mortality.

## Case presentation

**Figure 1:**
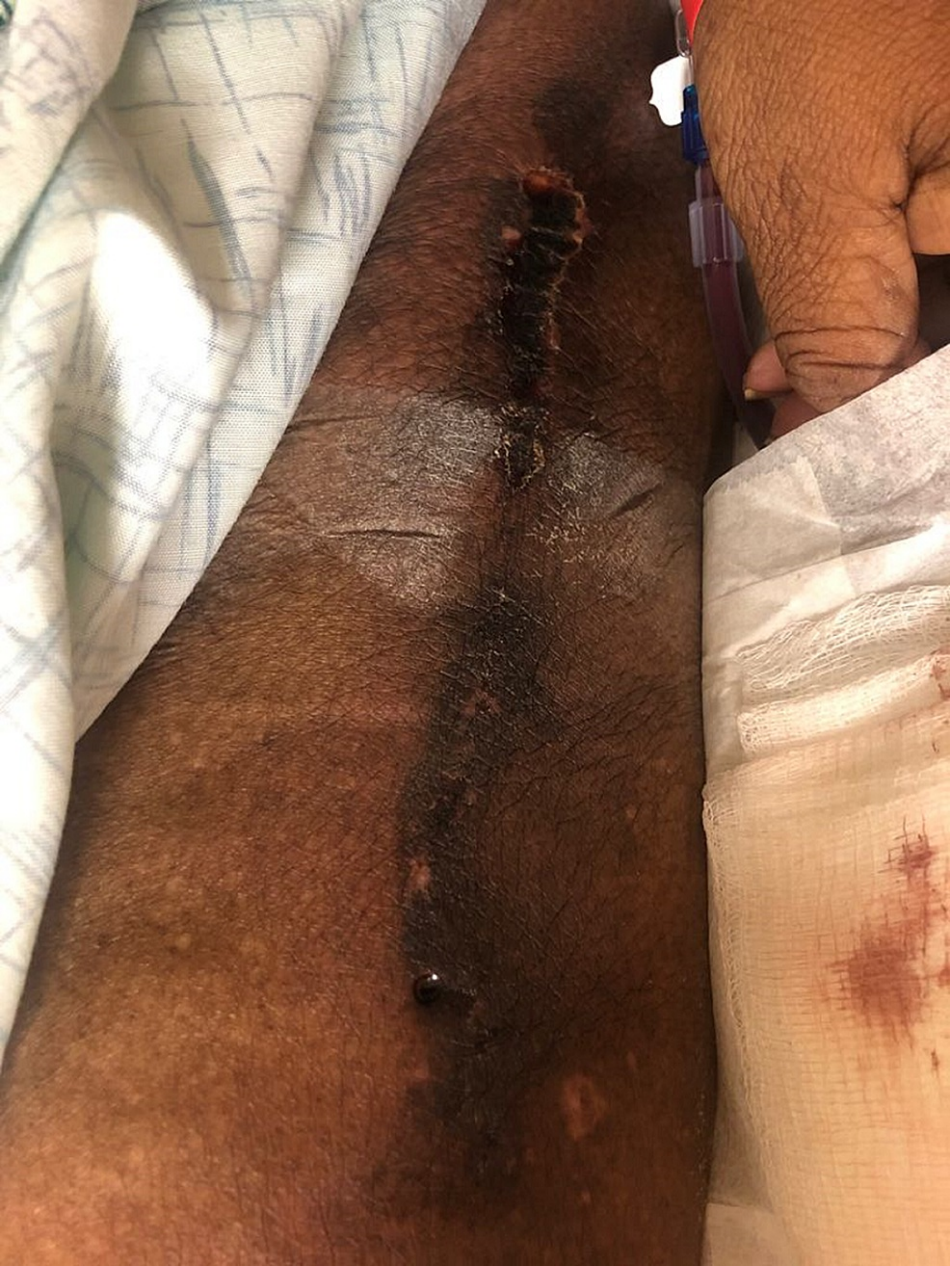
Left lateral thigh wound during initial admission

A 66-year-old African American woman presented to the hospital with shortness of breath, worsening pain, and swelling in the legs. Her medical comorbidities were significant for obesity (with BMI >40), diabetes mellitus type 2, hypertension, hyperlipidemia (HLD), gout, ESRD on hemodialysis (HD) for more than two years, warfarin use for arterio-venous graft clotting in the past, endometrial cancer and iron deficiency anemia. On examination, she had a longitudinal necrotic, indurated wound on the left lateral thigh which was tender and warm to touch with slight erythema (Figure 1). Another indurated wound was noted on the right medial thigh. She had these wounds for a few months, however, she did not seek any medical treatment. She had multiple admissions in the past for swelling in the legs and wounds on the lower extremities and was treated with antibiotics for possible cellulitis. She was admitted for wound infection management. 

Her labs were significant for an elevated white blood cell count of 20,100, anemia with hemoglobin 8.6 g/dL, serum creatinine 11.4 mg/dL, parathyroid hormone (PTH) level of 634 pg/mL, calcium 11.9 mg/dL, and phosphorus 10.7 mg/dL (she was not on any phosphate binders on admission). She was empirically started on IV vancomycin for possible cellulitis. A wound care specialist consultation was sought and the left leg wound was debrided and wound cultures were taken. There was a high suspicion of calciphylaxis, given her history of ESRD on dialysis, hyperparathyroidism, warfarin use, hyperphosphatemia, hypercalcemia, and female sex. A skin biopsy was performed. Warfarin was discontinued as it is one of the strong risk factors for calciphylaxis. Aggressive intervention for her hyperphosphatemia with a phosphate binder (sevelamer carbonate) was started. The skin biopsy showed calcified vessels, focal necrosis, and acute inflammation, confirming calciphylaxis. The wound was confirmed to have a polymicrobial infection and was covered with appropriate antibiotics. She was stable for discharge to rehab with sodium thiosulphate therapy three times a week with dialysis. Hyperbaric oxygen therapy was recommended but was declined.

**Figure 2:**
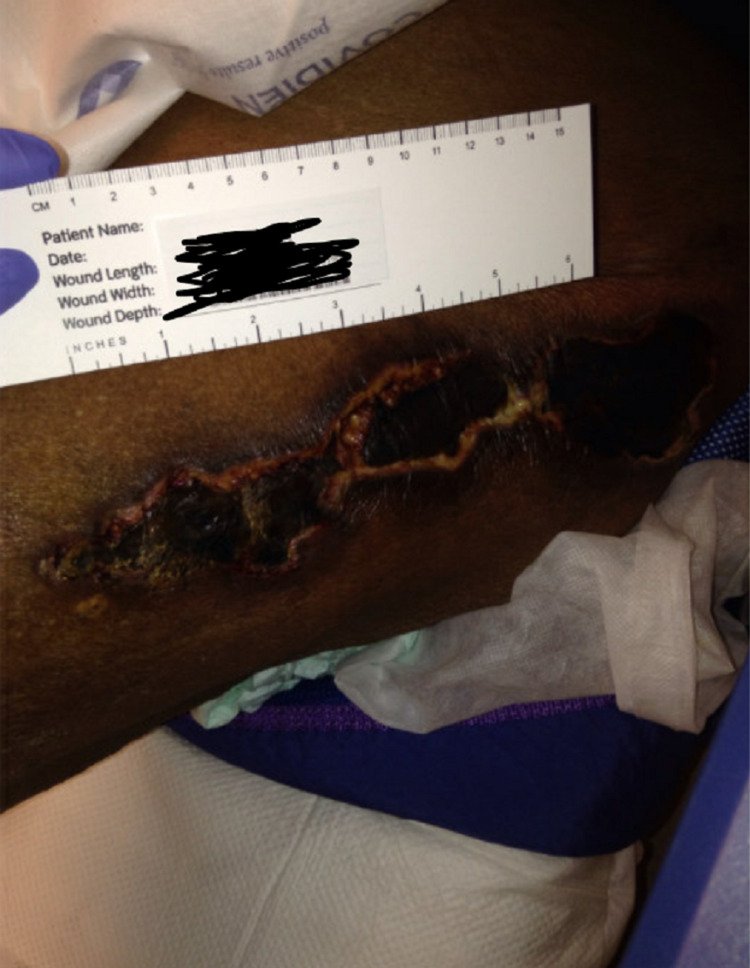
Left lateral thigh wound in the wound clinic after discharge from the hospital

**Figure 3:**
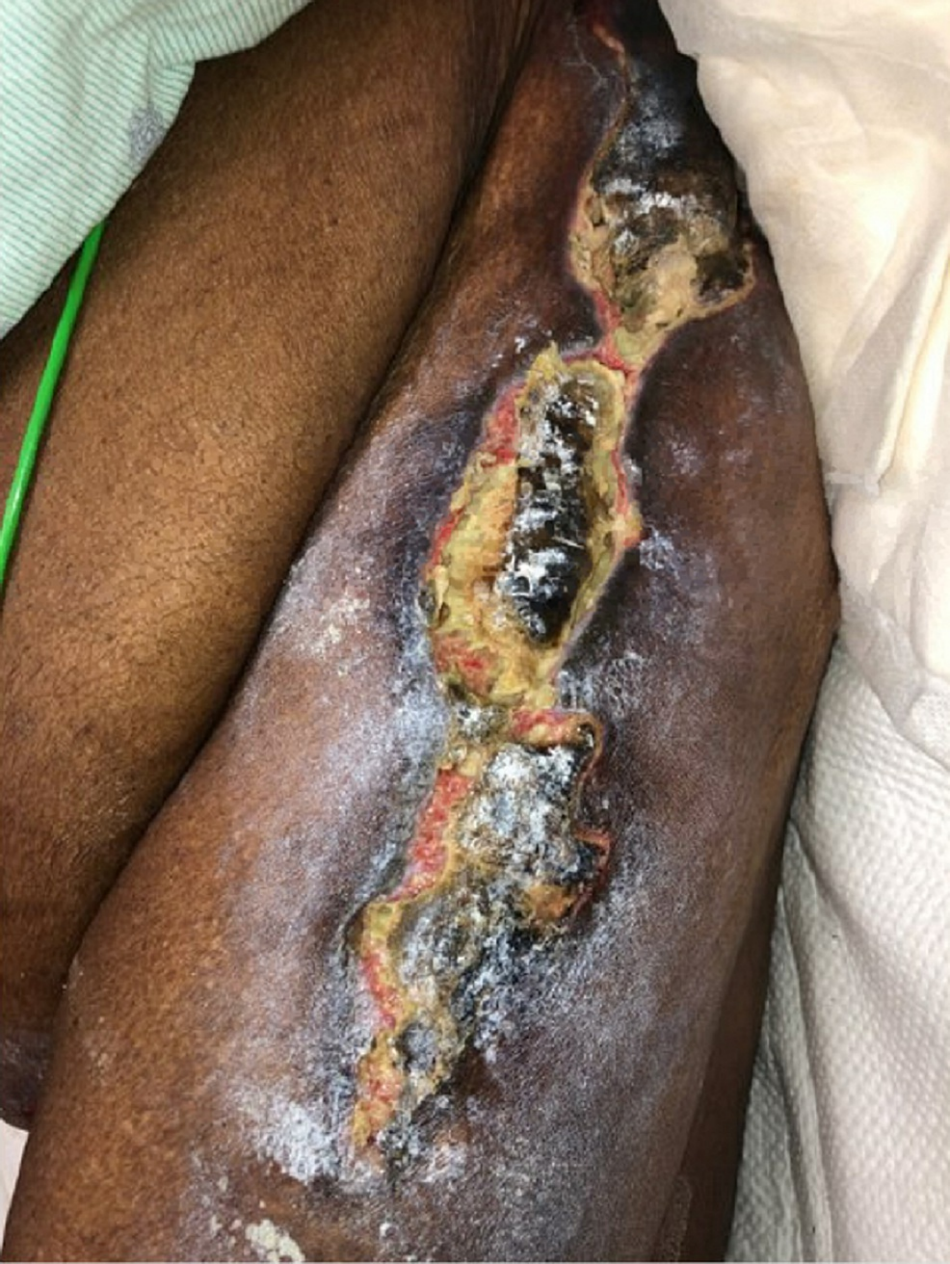
Left lateral thigh wound during the second admission

**Figure 4:**
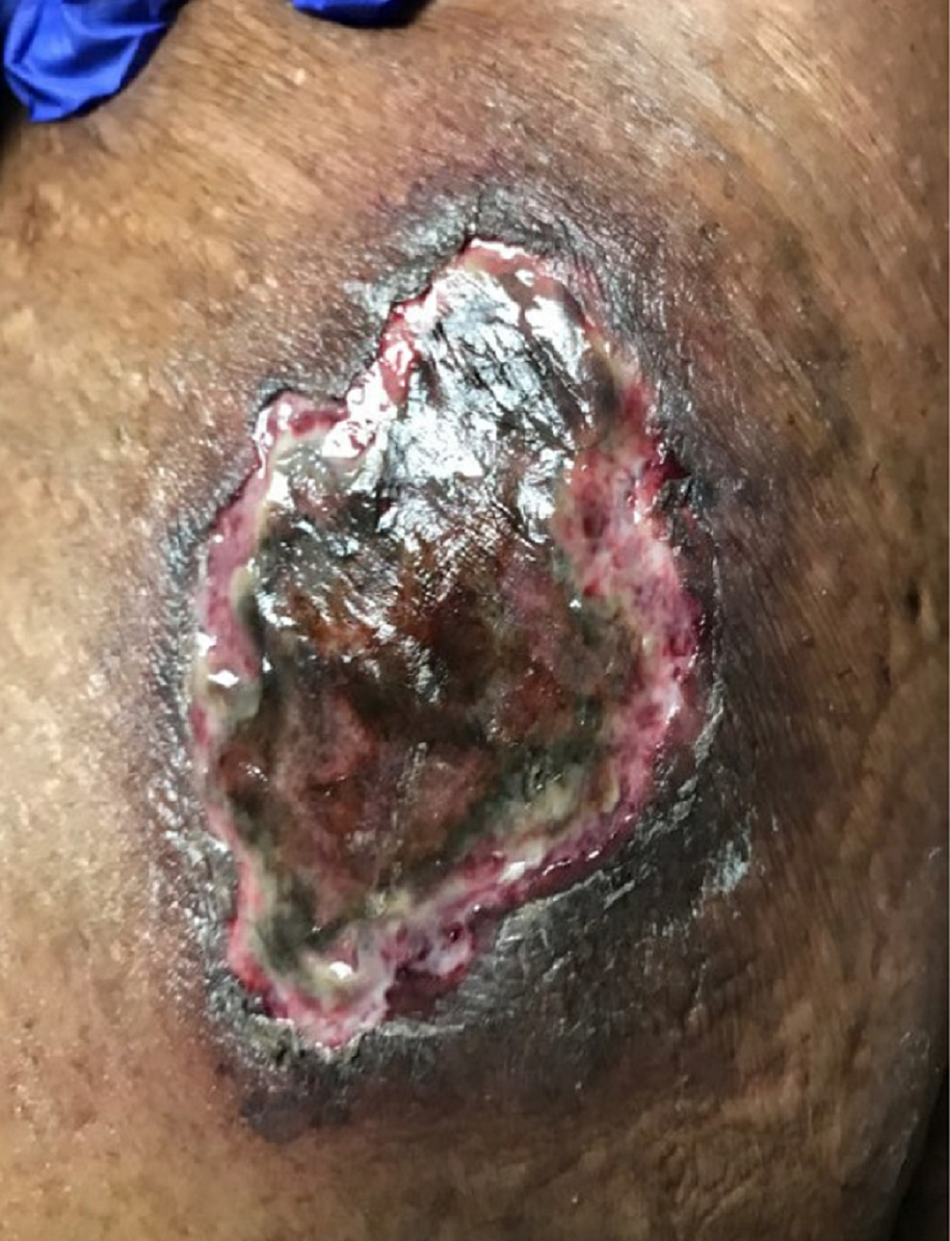
Abdominal wound with black eschar during the hospital readmission

Two weeks later, the patient was followed up in the wound clinic with slight worsening of the wounds and appropriate debridement was performed (Figure 2). She was readmitted to the hospital one month later, with altered mental status, acute respiratory failure with hypoxia, and multiple calciphylaxis wounds on the abdomen, legs, and right breast (Figures 3, 4).

Her wounds were adequately debrided and she was started on antibiotics. After some improvement, goals of care were discussed with the patient and she elected palliative and comfort measures with no further invasive surgical or medical interventions; she died later that month.

## Discussion

Calciphylaxis or calcific uremic arteriolopathy is a rare but life-threatening condition that is characterized by painful skin lesions including indurated plaques, livedo reticularis, and necrosis that can progress to penetrating ulceration with secondary wound infection. It is associated with high morbidity and mortality, with a reported one-year mortality of 30-80% [1]. Most commonly, it affects patients with ESRD and can occur in both peritoneal and HD patients. Risk factors include hyperphosphatemia, hypercalcemia, hyper- and hypoparathyroidism, hypercoagulability, diabetes mellitus, female sex, white race, autoimmune diseases, obesity, and warfarin use [2]. The pathogenesis of calciphylaxis remains unclear but microvascular calcification and thrombosis are considered likely processes in calciphylaxis development. Histopathologically, the pannicular small- and medium-sized arteries and arterioles typically show calcification of the media, intimal hyperplasia, endovascular fibrosis, and intravascular thrombosis resulting in an obliterative vasculopathy causing painful ischemic skin lesions [1,2].

Severe pain, which is believed to be both ischemic and neuropathic in origin, is a nearly universal feature of calciphylaxis and can sometimes precede the appearance of skin lesions [2]. It is characterized by non-healing wounds primarily involving the adipose-rich areas of the trunk, including breasts, flanks, lower back, and buttocks, as well as proximal lower extremities. Ulcerated lesions also demonstrate black eschar in many patients [3]. These lesions can progress to systemic infections if left untreated. Cutaneous complications are the patient’s primary concern in most cases but vascular calcification can also occur in skeletal muscle, lungs, brain, intestines, and other organ systems, suggesting a more systemic process [3,4]. 

Calcification is predominantly a clinical diagnosis although the definitive diagnosis of calciphylaxis requires a skin biopsy [4]. Non-invasive imaging tools such as plain x-rays or nuclear bone scans can also be used to aid in the diagnosis of calciphylaxis. The bone scan is positive when the tracer technetium 99m-medronic acid binds to hydroxyapatite crystals at the calcified areas in the dermis and subcutaneous fat [2].

Treatment of calciphylaxis requires a multidisciplinary approach involving nephrology, pain management, wound care, dermatology, plastic surgery, and palliative care. The wound care team should be consulted regarding the selection of dressings, chemical debriding agents, frequency of dressing changes, negative pressure wound care, as well as antibiotic administration when necessary [5]. Hyperbaric oxygen therapy may be considered for recalcitrant wounds [6]. Pain can be difficult to manage in patients with calciphylaxis and has a significant effect on the quality of life, therefore, multimodal pain control with analgesics with different mechanisms of action, biofeedback, and relaxation techniques should be used. Opioids can be used for pain control, but close monitoring is recommended, especially in dialysis patients where opioid drug levels are unpredictable and adverse effects, such as falls, are common. Palliative care should be involved to optimize end-of-life care, for symptomatic management of pain, lack of sleep, and appetite as well as for emotional/social/psychological support. Sodium thiosulfate has emerged as an important therapeutic option for the management of calciphylaxis. It was used initially for its calcium chelating properties, but it has been shown to have antioxidative and anti-calcification properties as well [7]. Serum calcium and phosphorous levels should be maintained in the normal range. Serum parathyroid hormone levels should be maintained between 150-300 ng/mL, and excessive suppression of parathyroid hormone should be avoided. For patients with poor wound healing and refractory mineral bone disease, the adequacy of dialysis should be investigated. Aggressive dialysis in the form of increased frequency or duration should be considered. Calcium supplements, high dialysate calcium baths, and vitamin D preparations should be avoided. Surgical parathyroidectomy is indicated in patients with refractory hyperparathyroidism but the studies showed controversies [8,9]. A nutrition consult should be obtained to address protein-energy malnutrition.

## Conclusions

Calciphylaxis is a rare but life-threatening condition commonly seen in patients with end-stage renal dialysis on renal replacement therapy. Diagnosis requires a high degree of suspicion based on clinical features and can be supported by skin biopsy. Physicians should be cautious when treating patients with ESRD for cellulitis and chronic wounds, and calciphylaxis should be in their differential diagnosis. Early identification of such patients is crucial to allow prompt treatment and to avoid the high morbidity and mortality associated with calciphylaxis.
